# Exploring the Success Factors for a Launch of an Algorithmic Consulting Platform

**DOI:** 10.1007/978-3-030-58858-8_7

**Published:** 2020-08-18

**Authors:** Andreas Kaselow, Dimitri Petrik, Sven Feja

**Affiliations:** 6 IT University of Copenhagen, Copenhagen, Denmark; 7 University of British Columbia, Vancouver, BC Canada; 8 Mercedes-Benz AG, 71063 Sindelfingen, Germany; 9 University of Stuttgart, 70174 Stuttgart, Germany; 10 adesso SE, 70173 Stuttgart, Germany

**Keywords:** Algorithmic consulting, Digital platforms, Consulting platforms

## Abstract

Even the rather traditional consulting industry is not spared from digitization. Digital platforms are known to foster convergence and generativity. For the consulting industry, digital platforms offer the potential to win new customer groups who have not previously purchased consulting services before. If digital platforms for the mediation of consulting services are established by incumbent consulting companies, the new platform-based business models for the consulting market will appear. Since network effects fuel platforms, the launch phase of a platform is a serious challenge. In this paper, we examine the launch of a digital platform for the algorithmic consulting (AC) approach, due to its promising market potential. In order to research the adaptability of digital platforms for AC consulting, we perform a qualitative study of electronic documents on the actions of three successful crowdsourcing platforms. The preliminary results comprise 14 success factors for the platform launch that will be validated in a follow-up practical application. The open nature of platform design in the areas of customer access, cooperation with other platforms, interfaces, and communication appeared to be particularly important for a successful platform launch. All identified success factors can be applied to AC services and the consulting market in the future.

## Introduction

Contributing a technical infrastructure, platforms usually connect independent companies, fostering generative activities [1]. Generativity is a characteristic of platform-centric markets, whereby digital technologies foster unprompted value creation through the recombination of various capabilities provided by independent companies [2]. Generativity reshapes markets and challenges the incumbent companies since the competition rules change [3, 4]. Platforms rely on the attraction of third-party companies, resulting in a platform-based ecosystem. Affected by network effects, the attractiveness of the platform and the aligning ecosystem are dependent on the number of complements, the number of complementors, and their value contribution [5].

In general, digitization opens up new possibilities for solving consulting-specific problems by handing them over to be solved by a large number of people with the appropriate expertise. This is exactly where the idea of consulting platforms comes into play, where agents with problems meet agents with solutions [6]. We are not aware of any integrative research on the platformization of the consulting business. While there are some case studies on digital consulting and consulting platforms in practice, the amount of scientific and academic literature is still rather small [7, 8].

Our paper analyses the application of platform-based business models to the consulting market using algorithmic consulting (AC) as an exemplary but promising digital business model in the consulting market. Market research in the German consultancy industry by Nissen et al. reveals that the supply of AC-related solutions is rather low, whereas the customer acceptance in contrast to other digital business model approaches is nearly the same [9]. Therefore, we conclude that the establishment of an AC consulting platform has the highest potential among all the consulting approaches [9]. Using secondary data, we apply a case study research design to research the question of *which success factors could help to implement the platform*-*based approach for algorithmic consulting business models*. For this purpose, we examine three existing platforms to extract success factors (SF) from their actions related to the dynamics of platform establishment. The preliminary results consist of 14 SFs regarding the platform launch, distributed over three levels of consideration. Further research on the validity of the derived SFs needs to be performed through a practical application by incumbent consulting companies. This article contributes to the body of knowledge on chicken-or-egg problems in platform context [10]. This problem is a significant challenge for new platforms to overcome, thus indicating the relevance of the conducted research when adding the perspective of incumbent consulting companies, who would like to establish platforms in the consulting market.

## Related Concepts

This paper draws on the theoretical concepts of digital platforms [11] and the concept of platform evolution [10]. In this section, we introduce the concepts of digital platforms, followed by a description of an algorithmic consulting business model to develop a theoretical pre-understanding of how platforms can facilitate the consulting market. Evans conceptualized platforms from an economic perspective, defining them as multi-sided markets to connect multiple sides of a market and manage their relationships, fostering generativity and convergence [11]. Similarly, a consulting platform primarily targets two customer segments, further described as seeker and solver. The seeker describes the customer segment that has a problem and is looking for a solution to reduce their costs, benefit from external expertise, or profit from external capabilities. In practice, seekers may be represented by big as well as small and medium-sized enterprises, startups, or private individuals. The customer segment of solvers is willing to offer solutions to the seeker problems. The solvers can be categorized as consultants, project managers, developers, or testers. Solvers seek to join the platform to acquire new projects and get in contact with new customers, for whom traditional consulting is too expensive. From the technological perspective, platforms usually have a modular architecture and are, therefore, extendable regarding their range of functions [10, 11].

Against this background, new consulting platforms are affected by the chicken-or-egg-problem as well. The establishment of a platform-based ecosystem may be constrained by the absence of seeking or solving customer segments [10].

*Algorithmic Consulting* is described by the automation of components of the consulting process. Especially data analysis components are suitable for algorithmic calculations to support the consulting process. The goal of AC is to substitute the consultant by algorithms that propose a solution for the customer. This enables scalability and leads to a consulting-based business model with exponential growth. For instance, algorithms can be used to analyze process data in transactional systems automatically and detect abnormalities. The AC software can trigger interventions when abnormalities are detected and then automatically generates reports and allocates decisional guidance. That is how AC shifts the consultant’s role into a supervisory role with the possibility to give further advisory support if needed. Another example are automated reports for executives based on structured data. These reports are able to determine and visualize trends and critical processes that can help to make faster decisions [12]. Further examples of suitable use cases can be found at McKinsey Solutions, which provides a collection of data-driven solutions in the context of AC [13]. An *AC platform* is characterized by a platform where different AC software and solutions are provided to solve customer problems with the use of algorithms as well as additional consulting services to support the customer.

## Research Approach

As mentioned in the first section, we chose three case studies to study the introduction of platforms to establish an AC platform in the market. The chosen companies are Upwork, Topcoder, and Innocentive. A brief overview of their financial metrics is depicted in Table 1, and the full list of used sources is available online at: https://bit.ly/2JRVAsx.

**Table 1.** A brief overview of the financial metrics of the studied platform companies

Platform	Profile	Year founded	Approx. revenue in 2019 (Mio USD)	Community size
Upwork	Focus on the mediation of freelancers and problem solvers	1999	300	14000000+
Topcoder	Focus on solving the problems in the software domain conducting challenges and tournaments	2001	19	1200000+
Innocentive	Focus on the solution of scientific problems, also organizing challenges and tournaments	2001	10	400000+

The reason to choose these three platforms can be explained with their success. During preliminary market research, we found that all three platforms represent successful platforms on the basis of key financial figures. All three platforms have successfully built multi-sided communities around their platforms. For instance, Topcoder still provides consulting services, but they established a platform-based business model in the area of crowdsourcing, matching solvers, and seekers in challenges for competitive programming. All of the examined companies offer at least a four-digit amount of challenges. Hence, certain levels of financial success and maturity make it possible to derive factors on how to establish a business model for platform-based AC for an incumbent consulting company.

Methodically we rely on the longitudinal case study analysis to track the platform evolution based on event streams related to a specific investigation object. The first step consists of the search and selection of publicly available secondary data resulting from the analysis of blog posts and press releases of the platform websites, technical blog websites, scientific articles, and archival versions of the platform websites. The examined articles, as well as the archived versions of platform websites, provided information on how the examined companies proceeded to launch a platform in their initial period. In addition, the timestamps of the analyzed articles helped to select only the articles about the initial phase. The analyzed dataset consists of 37 articles in a timespan between 2001 and 2020 (https://bit.ly/2JRVAsx). The second step is concerned with the content analysis of the data obtained to determine SFs. Thus, the data collection and the analysis approaches are inspired by Bowen’s document analysis. This qualitative research method helps to capture the context of the analyzed data, adding background, and historical insight [14]. Similarly, we use the contextual data of each platform to capture their specific contexts and understand how they succeeded to establish platforms and form communities. We derive the SFs by interpreting what we notice during the analysis of the secondary data sources. Additionally, the inclusion of additional and comparable case studies should ensure the completeness and generalizability of our interpretations of the SFs. The main difference between the approach in this paper and the case study conducted by Skog et al. [15] is that no phases are determined to explain the evolution of a digital platform over time, but SFs are explicitly identified for the introduction phase of a consulting platform. The introduction phase describes the entering of a market that follows the R&D phase and transitions into the ascent phase [10]. This difference was relevant in the analysis procedure since it is possible to extract phase-independent SFs from late phases that may be relevant for the introduction phase. Overall, we are confident that the utilization of secondary data in multiple specific contexts of each case study is suitable to generate valid insights, comparable with interviews or observations [14, 16].

To structure the content analysis in the electronic data, we used Tiwana’s vision of the evolutionary development of platforms due to its comprehensiveness and clustered the SFs in three levels of consideration: **strategy**, **architecture**, and **governance**. According to Tiwana, design and organization of platform ecosystems are affected by adapting these three dimensions [10, 17] and cover the aspects of an AC platform, being a modularly extendable software platform to mediate different parties (i.e., seekers and solvers). By assigning the actions of the observed platform companies to these three dimensions, we design a structured design framework on platform launches.

## Results

The following section provides an overview of the extracted SFs for a platform launch, which will be further described in more detail. The sources for each SFs, summed up as a table, are available online at: https://bit.ly/2JRVAsx.

**Strategy: (1)** The first SF suggests *Focusing on one specific value proposition* and building up expertise in that domain. Therefore, an owner of an AC Platform has to focus on the specific approach of AC and develop know-how in AC as well as Artificial Intelligence (AI). **(2)** One means for *Customer acquisition* describes the utilization of existing partnerships, publishing their AC-related challenges on the platform. This increases the number of seekers and challenges, which constitutes an incentive for solvers to migrate on the platform. In addition, partnerships to universities can be built to acquire students for challenge solving or to encourage them to develop and publish their own AC software on the platform. Another means includes the usage of internal consultants and developers with knowledge of AI (if available), who can be assigned to solve customer challenges on the platform. Other effective means are customer referral programs to subsidize the customer groups (solver & seeker) as well as online challenge-solving competitions to attract solvers to the platform. **(3)** *Customer loyalty* can be achieved by collecting and reacting to customer feedback. One means is to establish online forums where customers can write feedback articles about value proposition, price structures, or strategic decisions. A further means is founding a community advisory board (CAB), which consists of both customers and representatives of the platform to discuss improvement proposals. **(4)** *Marketing* provides means to use the existing communication channels (Social Media channels like Facebook, Youtube, Instagram etc.) to increase publicity and to get their followers to visit & join the platform. Also, the own corporate blog can be used to publish longer and more detailed insights of the technical functions as well as use cases of the platform. In addition, search engines can be used through adverts and keyword optimization to raise the traffic of the platform website. Moreover, exhibitions and events offer the opportunity to get physical contact with potential customers, where questions can be answered individually in person. **(5)** The *Expansion in emerging markets* intends to focus on one specific market where the platform owner is well informed at first. New markets can be explored and engaged afterwards. **(6)** *Openness* is meant to create an open strategic alignment of the platform, which impacts the dimensions of architecture and governance that need to follow this openness. One means is the definition of rules to steer the openness of the platform. The other means is the monitoring of whether the rules are obeyed. **(7)** The *Platform competition* recommends collaborating with other platforms to extend the value proposition by the value proposition of other platforms. This constitutes synergies between both platforms to gain more customers and extend the value proposition portfolio of the collaboration platform.

**Architecture: (8)** The *Selection of the value proposition* describes which propositions the AC platform should supply. The recommendations for the selected value proposition of the platform include an AC app store, the execution of AC-related challenges, and additional consulting services. The AC app store describes a marketplace offered as an online-website platform where solvers are able to supply their developed AC-Applications. The model for this app store is constituted by the web-based platform *Solutions* [13], which follows a closed design. Since the strategic advice is to follow an open approach, it is recommended to create an open design that allows easy usage of the applications through the online platform. The second value proposition describes the ability to publish challenges on the platform to solve customer-specific AC-related problems that are not covered by the available applications in the app store. The models for the challenge-based approach are Innocentive and Topcoder. It is conceivable that solutions could be hosted in the app store in a way to award the seeker & solver for future usage. This would mean an extension of the app store supply where the platform owner, as well as a new seeker, will benefit. The third value proposition is represented by additional consulting services, which can be provided by the consultants of the platform owner. One offered service could be assisting in identifying a suitable application within the app store, which fits the needs of the seeker precisely. Another consulting service could be defining and processing challenges in the name of the seeker. **(9)** *Modularity* means in the case at hand for the AC platform that the online-website constitutes the core component, whereas the AC-applications and services of the solver are the peripheral components. The premise for the integration of peripheral applications and services is an open platform architecture that allows solvers to offer their AC-related solutions via the online website. Therefore, open interfaces are required to ensure the technical integration of the solutions. **(10)** The results of the case studies showed that *Design* is an important function for the customers. Therefore, the UI-Design should be clear and able to be operated intuitively.

**Governance: (11)** The *Access* is influenced by strategic orientation and *Openness* (6). Therefore, the organizational restrictions imposed on access for new customers should be as low as possible. The case study analysis has shown that this factor directly helps to ensure critical customer mass. To fulfill these factors, possible means could include an easy registration process and importing customer data from other profiles (Google, LinkedIn, GitHub etc.). An additional means could be to design a short registration form with the possibility to provide necessary profile data after following the registration. Furthermore, an AC platform provider should not implement strict standards or requirements that would prevent new customers from joining the AC platform if they do not meet them. **(12)** *Interfaces* determine the extent to which solvers can integrate their own applications and services into the platform or the integration of platform functionalities into external applications and websites. A provision of a publicly available and well-documented API is required. Therefore, as with Upwork and Topcoder, an API library should be published in an easily accessible way on the online portal for all interested parties. **(13)** The P*rice structure* should be dependent on the customer segment. In customer acquisition, the focus is initially on the seeker, since solvers are initially available if an incumbent consulting company launches the platform. Therefore, in the introductory phase, the aim is to subsidize the seekers by waiving fees for the use of platform functions. This includes, for example, the costs for consulting services or fees for challenge tenders, which would have to be paid to the platform owner. The seeker only pays for the use or purchase of apps and services used by the solver, from which the payment is transferred in full to the solver. As an example, an AC platform company could charge solvers with commission fees from application purchases or challenge prizes. **(14)** *Communication* contributes to communicating information and decisions regarding the platform to customers. The existing social media and marketing channels, as well as the corporate blog of the incumbent company, can be used for this. A further means is having the founded CAB be in direct contact with customer representatives and to record their needs regarding platform relevant changes.

## Discussion

### Results

In conclusion, in this preliminary paper, we pursue the goal of identifying important SFs for practical application on the basis of real events. With this, we generated a generally applicable list of SFs for the platform launch, derived from actions of already established crowdsourcing platforms.

The identified SFs hold dependencies between each other. Generally, the strategic SFs have an effect on the design of the architectural and governance-related SFs. The distinction can be divided into sequential and technical dependencies. Sequentially, the SF *Openness* shapes the platform’s alignment to open up *Access, Interfaces, Communication,* and *Platform Competition* for the customer segments as well as other platforms to collaborate with. Technically, the *Modularity* of a platform’s architecture affects the *Selection of value proposition, Design, Access,* and *Interfaces* to facilitate the integration of components into the platform.

### Limitations

These findings, however, only allow us to conduct logical interpretations of the steps the three observed platform companies undertook. The results do not allow extracting cause-and-effect relationships between the actions and their effects in practice. For this step, more research is required in the area of ecosystems engineering [18] to identify the barriers of entry for solvers and seekers and to evaluate how practicable and comprehensive the derived SFs are. Moreover, the identified SF list does not hold the claim of completeness since the analysis is limited to the given data sources.

### Future Research

In the future, we will address this with a field study, applying the derived SF list to adesso SE, an incumbent consulting company, to help it to start a platform-based business model in AC. In addition to that, more research is required for the integrity of the identified SF list. For example, the quality of the engaged solver may have a significant impact on the success of a platform. However, it is not considered in the current state of the paper and has to be examined meticulously in the future. During this step, we will also pursue the goal of transforming the SFs into a domain-specific design framework to assist practitioners in the application of the identified SFs with guidance on best practice and implementation approaches. Furthermore, additional research is required to observe AC platform companies regarding how they implement the recommendations. The practical application of the derived SFs will help to review what cause-and-effect relationships they have to launch a successful AC platform. For instance, Upwork, which is the most successful platform in terms of sales and registered profiles, stands out due to its open nature in the areas of customer access, cooperation with other platforms, interfaces, and communication. *Openness* and its related SFs seem to play a more important role than the other extracted SFs. Therefore, we are convinced that these SFs, as well as Upwork need to be studied in more detail.
